# Improved Real-Time Influenza Surveillance: Using Internet Search Data in Eight Latin American Countries

**DOI:** 10.2196/12214

**Published:** 2019-04-04

**Authors:** Leonardo Clemente, Fred Lu, Mauricio Santillana

**Affiliations:** 1 School of Engineering and Sciences Tecnologico de Monterrey Monterrey Mexico; 2 Computational Health Informatics Program Boston Children's Hospital Boston, MA United States; 3 Department of Pediatrics Harvard Medical School Boston, MA United States

**Keywords:** google flu trends, influenza monitoring, real-time disease surveillance, digital epidemiology, influenza, human, developing countries, machine learning

## Abstract

**Background:**

Novel influenza surveillance systems that leverage Internet-based real-time data sources including Internet search frequencies, social-network information, and crowd-sourced flu surveillance tools have shown improved accuracy over the past few years in data-rich countries like the United States. These systems not only track flu activity accurately, but they also report flu estimates a week or more ahead of the publication of reports produced by healthcare-based systems, such as those implemented and managed by the Centers for Disease Control and Prevention. Previous work has shown that the predictive capabilities of novel flu surveillance systems, like Google Flu Trends (GFT), in developing countries in Latin America have not yet delivered acceptable flu estimates.

**Objective:**

The aim of this study was to show that recent methodological improvements on the use of Internet search engine information to track diseases can lead to improved retrospective flu estimates in multiple countries in Latin America.

**Methods:**

A machine learning-based methodology that uses flu-related Internet search activity and historical information to monitor flu activity, named ARGO (AutoRegression with Google search), was extended to generate flu predictions for 8 Latin American countries (Argentina, Bolivia, Brazil, Chile, Mexico, Paraguay, Peru, and Uruguay) for the time period: January 2012 to December of 2016. These retrospective (out-of-sample) Influenza activity predictions were compared with historically observed flu suspected cases in each country, as reported by Flunet, an influenza surveillance database maintained by the World Health Organization. For a baseline comparison, retrospective (out-of-sample) flu estimates were produced for the same time period using autoregressive models that only leverage historical flu activity information.

**Results:**

Our results show that ARGO-like models’ predictive power outperform autoregressive models in 6 out of 8 countries in the 2012-2016 time period. Moreover, ARGO significantly improves on historical flu estimates produced by the now discontinued GFT for the time period of 2012-2015, where GFT information is publicly available.

**Conclusions:**

We demonstrate here that a self-correcting machine learning method, leveraging Internet-based disease-related search activity and historical flu trends, has the potential to produce reliable and timely flu estimates in multiple Latin American countries. This methodology may prove helpful to local public health officials who design and implement interventions aimed at mitigating the effects of influenza outbreaks. Our methodology generally outperforms both the now-discontinued tool GFT, and autoregressive methodologies that exploit only historical flu activity to produce future disease estimates.

## Introduction

### Background

With the highest mortality of any respiratory infectious disease in the young and elderly in Latin America, influenza poses significant health and economic challenges to low- and middle-income countries in the region [1]. The World Health Organization (WHO) maintains a health care–based disease surveillance system that collects information on flu activity from local ministries of health around the world. Unfortunately, these reports have a common delay of at least a week in Latin America, limiting the ability for a timely response to unexpected epidemic outbreaks. Reliable surveillance systems that monitor flu activity in real time in this region would help public health institutions deploy timely vaccination campaigns and optimally allocate resources during epidemic outbreaks. Multiple research teams have proposed complementary methods to estimate and forecast flu activity in real time in data-rich countries such as the United States, using techniques ranging from statistical [2,3] to mechanistic [4,5] and incorporating a variety of data sources, such as internet search information, flu-related Twitter microblogs [6,7], crowdsourced flu surveillance [8,9], clinician search activity [10], electronic health records [11], and Wikipedia access [12,13], as summarized in a study by Santillana [14]. However, a reliable system that leverages internet search activity to monitor flu activity in multiple developing nations is not yet available.

An early large-scale implementation of real-time disease surveillance started in 2008 with Google Flu Trends (GFT), a Web-based tool that used Google search activity to produce flu activity estimates in multiple locations around the world [15]. Although GFT was initially perceived as a technological innovation, its large prediction errors during the 2009 H1N1 flu pandemic and the 2013 flu season in the United States raised methodological concerns from multiple researchers [16-18]. A recent study by Pollet et al showed that GFT’s flu estimates in Latin America had yielded poor results [19].

### Objectives

The discontinuation of GFT in 2015 led many to believe that internet search trends were too noisy to track disease activity, a problem exacerbated in developing countries with limited internet access. However, recent research has shown that robust and dynamically self-correcting machine learning methodologies can extract meaningful signals from real-time search activity to track Zika and Dengue activity in low- to middle-income countries around the world [11,20,21]. We apply lessons learned from these studies and successfully extend a state-of-the-art modeling approach for flu surveillance to 8 Latin American countries.

## Methods

### Data Acquisition

We built a predictive methodology aimed at estimating suspected flu activity, as reported by FluNet, an online surveillance tool maintained by the WHO. FluNet collects and aggregates multiple indicators of flu activity at the country level. For this study, we selected the number of processed specimens (NPSs) as the ground truth. As these specimens were taken from patients with flu-like symptoms and then sent to a laboratory for testing, we interpreted them as an indicator of suspected flu activity in the population. Weekly aggregated NPS reports were collected from January 5, 2009, to December 25, 2016, for Argentina, Bolivia, Brazil, Chile, Mexico, Paraguay, Peru, and Uruguay.

Given their near real-time availability via the online tool, Google Trends, we selected influenza-related internet search activity to be used in our models as proxies or predictors for flu activity. Where available and based on country-specific historical flu indicators (during the training time period of our models), we used the online tool, Google Correlate, to identify flu-related search term trends, leading to a total of 285 Spanish terms and 96 Portuguese terms. See Multimedia Appendix 1 for further description of this process.

### Models and Benchmarks

We extended AutoRegression with Google search information (ARGO), a methodology originally conceived and tested to track flu activity in the United States in multiple spatial scales as a way to produce retrospective and strictly out-of-sample flu estimates individually for each country [20,22]. This methodology is based on a multivariable regularized linear model that is dynamically recalibrated every week as new flu activity information becomes available. Besides online search information, ARGO incorporates short-term and seasonal historical flu information to improve the accuracy of predictions and mitigate the undesired effect of spikes in search activity (induced perhaps by overreaction in the population during potential health threats reported by the news). More details on this approach can be found in a study by Yang et al [22].

Given a weekly as-yet-unseen NPS report to estimate, we used historical NPS and Google Trends information from the previous most recent 2 years (104 weeks) of data to calibrate ARGO and predict the given week’s NPS report. To assess ARGO’s predictive power, we built autoregressive models separately for each country (named AR52 throughout this paper) that only use historical flu activity from the 52 weeks before predictions and generated retrospective out-of-sample estimates over the same time period. All models were built using the glmnet package on MATLAB (MathWorks) version 2014a [3,23].

### Metrics

To compare the predictive ability of ARGO and AR52, we calculated Pearson correlations and the root mean square error between model predictions and the subsequently observed suspected flu cases. The added value of using Google search activity as a predictor was tested via an efficiency metric [22] that quantifies the improvement of ARGO over a simple autoregressive model. This efficiency metric is calculated as the ratio between the mean square errors of AR52 and ARGO. For the efficiency metric, 90% CIs were generated using the stationary block bootstrap method [24]. We report 2 additional metrics aimed at evaluating our method’s ability to correctly identify the timing of peaks and timing of the onset of epidemic outbreaks. These metrics are referred to as ∆P and ∆O, respectively, and measure the distance, in weeks, between the observed peak (or onsets) and the predicted one. See Multimedia Appendix 1 for further explanation.

## Results

### AutoRegression With Google Search Models Outperform AR52 and Google Flu Trends

Retrospective out-of-sample estimates of flu activity were produced, for each of the 8 countries, from January 1, 2012, to December 25, 2016, and compared with the FluNet reported suspected cases (NPSs). Brazil’s NPS data were only available until October 9, 2016. Note that because of FluNet’s reporting delays, our models, which rely on past available values of FluNet and current internet search activity, estimate current flu activity at least 1 week ahead of official reports.

**Figure 1:**
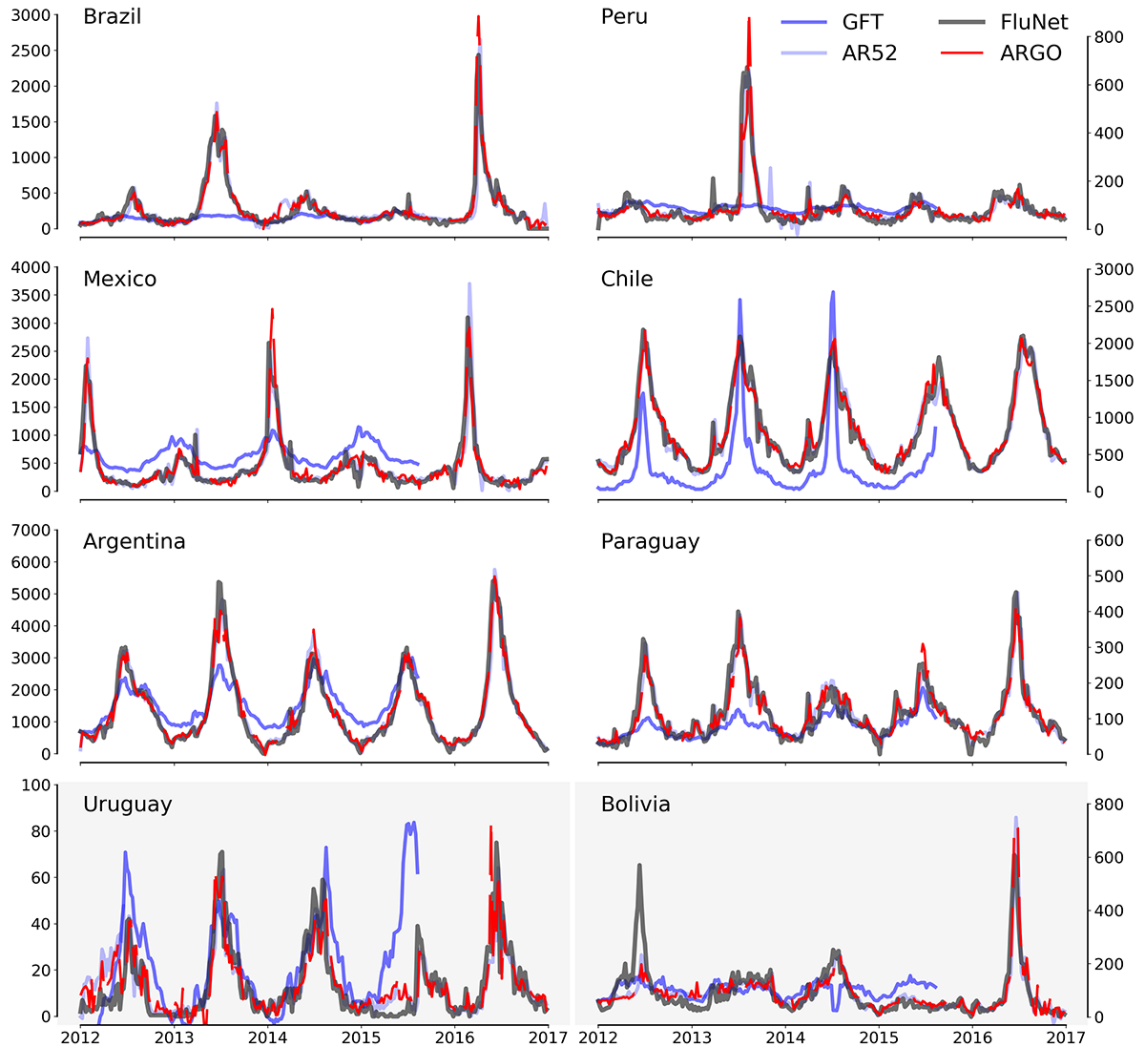
Graphical representation of the number of processed specimens (NPSs) as reported by WHO’s FluNet (black), along with the NPS estimates generated by ARGO (red), AR (light blue), and Google Flu Trends (GFT; blue), over the whole study period of January 1, 2012 to December 25, 2016.

In Figure 1, we show our real-time flu estimates and the subsequently observed suspected flu cases for each country. Contextually, historical GFT values (scaled to be displayed alongside with NPS values) and autoregressive estimates are also shown. Our models (ARGO and AR52) accurately predict NPS values in each country. GFT shows consistently large discrepancies when compared with the observed values, consistent with the findings reported by Pollett et al [19].

**Figure 2:**
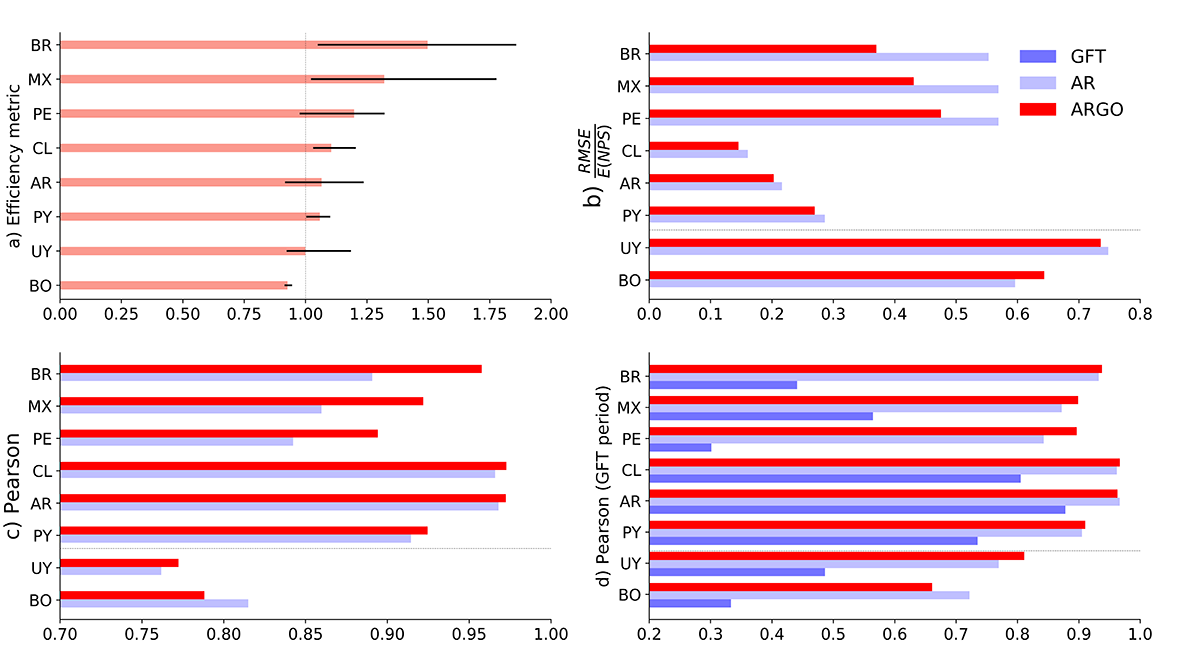
Set of bar graphs that show our performance metrics to assess the predictive power of AutoRegression with Google search (ARGO). (a) Efficiency metric values (salmon color) for each individual country with their respective 90% CI (solid black line). (b) Root mean square error (RMSE) values for ARGO (red) and AR52 (light blue) during the whole study period. Each country’s RMSE value is normalized by their respective average number of processed specimen (NPS) over the whole study period to avoid scale differences in visualization. c) Pearson correlation scores of ARGO and AR during the full period. d) Pearson correlation values for ARGO (red), AR (gray), and Google Flu Trends (GFT; blue) during the period in our study where GFT was active.

ARGO displays improvement in 6 countries in terms of the efficiency metric (Figure 2), reaching significant error reductions compared with AR52 in Brazil (155 to 104 or 33%), Mexico (243 to 184 or 24%), Peru (48 to 40 or 16%), and Chile (131 to 119 or 9%). ARGO consistently outperforms GFT on Pearson correlations during the time period when GFT was active in every country and improves upon AR52 in all countries except Bolivia and Uruguay, over the whole study period, reaching significant correlation increases in Brazil (from 0.891 to 0.957), Mexico (from 0.86 to 0.92), and Peru (from 0.84 to 0.89).

### Peaks and Onsets

Multimedia Appendix 1 shows our method’s ability to identify the timing of peaks and timing of the onset as captured by the metrics ∆P and ∆O, respectively. Out of a total of 39 peaks, ARGO predicts 10 on the observed week, 5 ahead of time, and 24 with a lag. In contrast, AR52 predicted 7 peaks exactly, 2 ahead of time, and 30 with a lag. Out of 39 measured onsets, ARGO predicted 11 at the exact date, 12 ahead of time, and 13 with a lag, with the rest of the onsets incorrectly estimated with more than a month’s distance. AR52 predicted 8 onsets at the exact time, 8 ahead of time, 20 with a lag, and the rest were estimated with more than a month’s distance. As expected, the baseline autoregressive model, AR52, predicted more outbreak peaks late, with a lag of at least a week. ARGO, in contrast, is slightly more responsive and predicts outbreak peaks on time or weeks before the observed peak. Furthermore, even when ARGO’s peak timing is not accurate, the magnitude of the peak is captured better than its AR52 counterpart. For more detailed information, see Multimedia Appendix 1.

## Discussion

### Combining Historical Flu Activity and Google Search Data

ARGO’s prediction performance shows that internet search volumes and historical flu activity, when combined with dynamic machine learning techniques, can effectively detect real-time suspected flu cases in several Latin American countries. Our results considerably outperform the historical predictive performance of GFT highlighting (1) the importance of moving away from one-size-fits-all approaches such as those used by GFT and (b) the value of combining local flu epidemiological information with influenza-related internet search trends. The overall improvement of ARGO over the baseline autoregressive model indicates that internet search engine data, even in middle-income countries, provide increased responsiveness to changing disease trends. This improvement is clear in Brazil, Chile, Mexico, Peru, Paraguay, and Argentina, whereas in Uruguay and Bolivia, the inclusion of Google search data does not seem to improve the baseline model.

The availability of an online tool to select relevant flu-related terms (Google Correlate) that track historical flu activity was found to be a critical element for ARGO to improve performance over the autoregressive benchmark (Argentina, Chile, Mexico, Peru, and Brazil), suggesting that the most meaningful flu-related search queries are country-specific. In countries, such as Uruguay, where many weekly data points were missing on FluNet, ARGO’s predictive ability was reduced. Our best performance was seen in Brazil, Mexico, and Peru, where flu data were collected consistently every week during this study’s time period (see Multimedia Appendix 1).

### Number of Processed Specimens as Our Gold Standard

On the basis of our previous research findings monitoring Dengue and Zika activity in Latin America [20,21], we chose the number of suspected influenza cases (as captured by the FluNet’s NPSs) as our gold standard for our prediction tasks. Our choice was based on the intuitive fact that flu-related Google search activity is higher when more people “suspect” they may be affected by flu-like symptoms, regardless of the outcome of any lab test. As such, our models may prove useful to improve the timely allocation of resources in health care facilities in situations when increased numbers of people, with flu-like symptoms and respiratory needs, may need to be seen. It is relevant to point out that using NPS case counts as a gold standard implies that our models are not directly estimating confirmed influenza case counts but suspected Influenza-like Illness activity trends. Our choice of gold standard is meaningful as it may help health care providers prepare for traffic fluctuations of patients presenting with symptoms of influenza. However, from an epidemiological perspective, more standard test-positive influenza proportions reported on previous Latin America studies [19,25] should also be considered in future studies.

### Limitations and Future Work

Combining internet search volumes and historical flu activity via ARGO shows strong potential for the development of timely flu surveillance in low- or middle-income countries. However, flu estimates at the national level may not be reflective of outbreak conditions at the local level, especially in countries with significant geographical heterogeneity. At present, FluNet only provides national-level flu data. In the future, as more fine-grained epidemiological information becomes available in developing countries, studies should evaluate the feasibility of deploying disease surveillance platforms at finer spatial scales. Successful extensions of our methodologies at the city and state levels in data-rich environments such as the United States [26,27] indicate that ARGO-like methodologies can accurately monitor influenza at these spatial resolutions. Moreover, there is strong evidence that internet access is rapidly increasing in many Latin American countries [28], leading us to hypothesize that the performance of methodologies using internet-based data will increase over time following an increase of the quality of Google search data.

## Appendix

Multimedia Appendix 1 Supplementary materials. This document includes several Tables and Figures, as well as more information regarding our Methods and Results section.

## Abbreviations

ARGO: AutoRegression with Google search
GFT: Google Flu Trends
NPS: number of processed specimens
WHO: World Health Organization
