# Supplementary material for: Improved Real-Time Influenza Surveillance: Using Internet Search Data in Eight Latin American Countries
Source: JMIR Public Health Surveill. 2019 Apr 4;5(2):e12214. doi: 10.2196/12214 (PMC6470460; doi:10.2196/12214)
Supplement: Multimedia Appendix 1 [file publichealth_v5i2e12214_app1.pdf]

Supplementary Materials

Data sources

FluNet

FluNet is a worldwide surveillance tool created in 1997 by the World Health Organization to track influenza activity. It provides country-level information regarding the number of reported, tested, and confirmed influenza cases, split by influenza subtype and reported in different table formats. FluNet's aggregated weekly reports for the eight countries were retrieved for the period of Jan. 5, 2009 to Dec. 25, 2016. From these tables, the total number of processed specimens (NPS) was used as the estimation target. Our choice of target is addressed in the Discussion section. Aside from adding new report data every week, FluNet also performs retrospective changes to previously reported data, a factor that may affect reproducibility.

Google Correlate and Google Trends

Google Trends is an online service that provides search frequency time series for any query documented in Google's search database. Google Correlate returns the most correlated search queries to any user-submitted time series. A general list of terms was created by:

1. Using FluNet's NPS time series as input in Google Correlate for Argentina, Brazil, Chile, Mexico, and Peru (Google Correlate is not available for Bolivia, Paraguay and Uruguay) and extracting the top correlated terms for dates prior to the study period.
2. Repeating this process with the time series for the search term "influenza" from each country.
3. Repeating this process with the most correlated term from the list obtained in step 1.

A separate list was generated for Brazil due to language difference. These lists were used to download Google Trends data for all the countries from Jan. 5, 2009 through Dec. 25, 2016.

Model

An ARGO (AutoRegressive model with GOogle search queries as exogenous variables) model was developed for each country to predict NPS activity one week ahead of FluNet's surveillance reports. The training set for each model consisted of the most recent 104 weeks of data prior to the desired weekly estimation. The use of dynamic time windows allows ARGO to re-calibrate to the most recent data and reduce overshooting.

Formulation

In the ARGO model, higher search frequencies for disease-related Google queries are observed when the disease has a higher impact, such as when people are infected or experience symptoms. NPS is modeled using the observed Google search frequencies and the flu case reports from past observed data as follows:

$$\hat{y}_t = u_y + \sum_{j \in J} \alpha_j y_{t-j} + \sum_{k \in K} \beta_k X_{k,t} + \epsilon_t, \epsilon_t \sim N(0, \sigma^2) \quad (1)$$

where:

- $\hat{y}_t$ is our estimate of NPS at time t

- y_{t-j} is the NPS observed at time $t-j$
- J is the set of autoregressive lags
- K is the set of Google query terms
- $X_{k,t}$ is the Google search frequency of term k at time t
- u_y is an intercept term.

The endogenous and exogenous variable coefficients $\alpha_j, j \in J$ and $\beta_k, k \in K$ were fitted using multivariable linear regression with L1 regularization (LASSO) and 10-fold cross validation to determine the level of regularization. The regression was re-trained on a weekly basis, updating the input features with the newest available data from FluNet and Google for the next prediction. This approach allows for recalibration of regression coefficients in a way that adjusts the variables based on their prediction ability over the training set.

Benchmarks

Two benchmarks were used to assess ARGO's performance. These are:

Autoregressive model

An autoregressive model uses as input only past values (i.e. lags) from the NPS timeseries. Using the previous notation, then

$$\hat{y}_t = u_y + \sum_{j \in J} \alpha_j y_{t-j} + \epsilon_t, \epsilon_t \sim N(0, \sigma^2) \quad (2)$$

For each country, we fit an L1-regularized multivariate linear model with 52 time lags, denoted AR52.

Google Flu Trends

Weekly flu activity values from Google Flu Trends (GFT) were collected from January 2, 2011 through August 9, 2015 (when GFT was discontinued). Google did not scale the values to any known official flu curve, so we rescaled the values to the FluNet NPS using a linear regression over the full study period.

Metrics

The models were used to retrospectively simulate real-time estimates of influenza NPS from January 1, 2012 through December 25, 2016. In the case of Brazil, NPS data was only available until October 9, 2016. Two accuracy metrics were used for model comparison:

1. Root mean square error: $\text{RMSE} = \left[\frac{1}{N}\sum_{t=1}^{N}(\hat{y}_t - y_t)^2\right]^{1/2}$

2. Pearson correlation coefficient.

The RMSE and Pearson correlation were calculated between each model and the FluNet NPS annually, over the entire prediction period, and over the sub-period when GFT was published (1/1/12 to 8/9/15). In addition, we calculated the inverse of the mean square error ratio between ARGO and AR (called the efficiency metric from now on) for the whole study period. Weeks for which the NPS activity were not available in each country were removed prior to computation of metrics.

Peaks and Onsets

The following metrics were defined and used to evaluate the ability of a model to correctly predict the timing of peaks and onsets of an epidemic outbreak:

1. Onset timing: Distance, in number of weeks, between the ground truth's outbreak onset and the model's estimated onset:
$$\Delta O = O_{model} - O_{observed}$$

2. Peak timing: Distance, in number of weeks, between the ground truth's outbreak peak and the model's estimated peak:
$$\Delta P = P_{model} - P_{observed}$$

Influenza outbreak onsets were empirically identified for each country as follows:

1. For a given country, we extracted each of the five years' epidemic outbreaks, consisting of 52 data points per year (see Figure S2).

2. On a single 52 week interval, we created a new curve by adding all of the five years of epidemic data.

3. Using the resulting curve, we found the threshold value that separates low activity from outbreak activity as the average NPS case count from week n such that, if we sum the average NPS case counts from week 1 to week n, the sum would represent 10% of the total sum over the 52 weeks of the curve. The threshold was then normalized (divided by 5) to be applied for each individual flu season.

4. Finally, for each outbreak, we identified the onset week as the first week when this threshold was crossed.

.

Supplementary figures and tables

Table S1: Pearson correlation for each model and country. The top performer in each time period is shown in bold. To allow comparison with GFT, correlation during Y2015 only included the period before it was discontinued (9th of August of 2015).

		Whole period	GFT period	Y2012	Y2013	Y2014	Y2015*	Y2016
Brazil	ARGO	**0.958**	**0.938**	**0.910**	0.953	0.705	0.441	**0.984**
	AR52	0.891	0.933	0.825	**0.957**	0.666	0.202	0.827
	GFT	–	0.441	0.625	0.766	**0.849**	**0.580**	–
Mexico	ARGO	**0.922**	**0.899**	**0.948**	0.790	**0.874**	0.890	**0.972**
	AR52	0.860	0.872	0.919	0.585	0.862	0.879	0.842
	GFT	–	0.565	0.531	**0.835**	0.617	**0.928**	–
Peru	ARGO	**0.894**	**0.897**	0.580	**0.904**	**0.694**	0.837	**0.814**
	AR52	0.842	0.843	**0.657**	0.843	0.618	**0.849**	0.804
	GFT	–	0.301	0.431	0.406	0.625	0.770	–
Chile	ARGO	**0.973**	**0.967**	**0.976**	0.963	**0.967**	**0.976**	**0.991**
	AR52	0.966	0.962	0.966	**0.965**	0.961	0.967	0.981
	GFT	–	0.805	0.888	0.850	0.809	0.940	–
Argentina	ARGO	**0.972**	0.963	0.963	0.968	0.965	0.968	**0.990**
	AR52	0.968	**0.966**	**0.969**	**0.975**	**0.966**	**0.979**	0.969
	GFT	–	0.878	0.919	0.938	0.937	0.971	–
Paraguay	ARGO	**0.925**	**0.911**	**0.928**	**0.927**	0.866	**0.914**	**0.956**
	AR52	0.914	0.905	0.918	0.920	0.822	0.874	0.931
	GFT	–	0.735	0.885	0.915	**0.879**	0.912	–
Uruguay	ARGO	**0.772**	**0.811**	0.628	**0.892**	0.857	0.050	0.681
	AR52	0.762	0.769	0.404	0.889	**0.894**	-0.072	**0.756**
	GFT	–	0.486	**0.811**	0.869	0.709	**0.183**	–
Bolivia	ARGO	0.788	0.661	0.584	0.675	0.914	0.605	**0.939**
	AR52	**0.815**	**0.722**	**0.688**	**0.789**	**0.923**	**0.630**	0.931
	GFT	–	0.333	0.641	0.607	0.001	0.610	–

Table S2: Root mean square error for each model and country.

		Whole period	GFT period	Y2012	Y2013	Y2014	Y2015*	Y2016
Brazil	ARGO	**104.38**	**94.78**	**53.78**	133.54	**92.90**	**72.52**	**155.91**
	AR52	155.87	101.03	72.23	**133.27**	101.94	76.81	316.27
Mexico	ARGO	**184.66**	**200.96**	**164.15**	**131.48**	312.47	**84.52**	**146.93**
	AR52	243.79	213.51	210.9	176.95	**290.15**	88.97	361.52
Peru	ARGO	**40.31**	**45.59**	24.83	**76.16**	**28.82**	21.89	**23.46**
	AR52	48.27	55.18	**23.62**	94.83	35.39	**20.36**	24.16
Chile	ARGO	**119.28**	**127.28**	**112.60**	141.74	**138.39**	104.47	**84.59**
	AR52	131.74	133.66	130.51	**138.86**	147.10	**103.96**	116.43
Argentina	ARGO	**274.12**	292.42	229.56	387.18	**265.30**	244.52	**217.92**
	AR52	292.01	**277.59**	**210.57**	**355.30**	289.78	**200.17**	376.24
Paraguay	ARGO	**30.81**	**31.37**	**29.19**	**36.50**	**28.83**	**29.68**	**31.75**
	AR52	32.679	32.30	29.55	36.73	31.65	29.88	38.10
Uruguay	ARGO	**10.01**	**9.27**	**11.07**	**8.91**	8.36	**8.41**	13.14
	AR52	10.17	10.32	14.10	8.92	**7.24**	8.57	**11.14**
Bolivia	ARGO	58.62	60.27	103.54	36.34	27.58	**17.88**	62.24
	AR52	**54.30**	**56.01**	**96.20**	**32.53**	**25.79**	19.91	**57.08**

Table S3: Efficiency metric for each country with 90% confidence intervals generated with the stationary block bootstrap. Scores above 1 indicate ARGO incurred less error than AR52.

Country	Efficiency	5th percentile	95th percentile
Brazil	1.497	1.050	1.858
Mexico	1.320	1.023	1.778
Peru	1.197	0.976	1.322
Chile	1.104	1.031	1.205
Argentina	1.065	0.916	1.237
Paraguay	1.058	1.003	1.101
Uruguay	0.999	0.923	1.185
Bolivia	0.926	0.914	0.945

Table S4: Search term list.

a h1n1, acemuk, acemuk 600, acemuk dia y noche, acemuk jarabe, acemuk l, acetilcisteina, ah1, ah1n1, ah1n1 wikipedia, alcanfor propiedades, alcohol en gel, alcohol gel, alerta epidemiologica, aliviar la tos, amoxidal, amoxidal duo, antitusivo, antiviral, antivirales, athos jarabe, atrovent, azitromicina, barbijo, barbijo 3m, barbijos, barbijos 3m, barbijos descartables, benadryl antitusivo, betametasona gotas, bisolvon pediatrico, bronconeumonia, bronquitis, bronquitis es contagiosa, bronquitis obstructiva, bronquitis sintomas, budesonida, budesonide, bufanda, calmar la tos, casos de influenza, catarro, causa de la influenza, causas de la gripe, cdc, cdc atlanta, cdc en espanol, cdc.gov, cebolla para la tos, cerco sanitario, claritromicina, cofron, combatir la gripe, como aliviar la tos, como calmar la tos, como curar la tos, como hacer alcohol en gel, como parar la tos, como prevenir la gripe, como se contagia la gripe, con gripe, con tos, congestion nasal, contra gripe, cronopen, cronopen balsamico, cubreboca, cubrebocas, curar la tos, decidex, decidex compuesto, decidex plus, definicion de pandemia, dolor de garganta, el catarro, el virus de la gripe, el virus de la influenza, emergencia sanitaria, enfermedad de la influenza, enfermedad influenza, epidemia, epidemia pandemia, epidemia y pandemia, estupefaciente, expectorante, expectosan, fiebre española, fiebre porcina, flemas, flu, flu symptoms, gripa, gripe, gripe a, gripe a en argentina, gripe ah1n1, gripe comun, gripe en el embarazo, gripe espanola, gripe española, gripe estacional, gripe h1n1, gripe o influenza, gripe porcina, gripe sintomas, gripe viral, gripes, grippe, h1n1, h1n1 en mexico, h1n1 influenza, h1n1 mexico, h1n1 sintomas, h1n1 wikipedia, imagenes de la gripe, influenza, influenza 2009, influenza a, influenza a h1n1, influenza ah1n1, influenza chile, influenza en chile, influenza en mexico, influenza equina, influenza española, influenza estacional, influenza estacionaria, influenza h1n1, influenza humana, influenza mexico, influenza porcina, influenza sintomas, influenza tratamiento, influenza virus, inmunodeprimidos, ipratropio, jabon gel, jarabe para la tos, la bronquitis, la bronquitis es contagiosa, la epidemia, la gripe, la gripe a, la gripe ah1n1, la gripe comun, la gripe h1n1, la gripe porcina, la influenza, la influenza a h1n1, la influenza ah1n1, la influenza en mexico, la influenza h1n1, la influenza humana, la influenza porcina, la neumonia es contagiosa, la pandemia, la porcina, la sinusitis, la tos, letondal, mascarilla n95, medicamento para la gripe, medicamentos para la gripe, medico a domicilio, mocos, mocos amarillos, mocos verdes, mucha tos, muco dosodos, mucolitic antitusivo, mucolitico, mucoprednibron, mucosolvan, muxol, n1, n1h1, nebulizador a piston, nebulizar, neumonologo, neuraminidasa, noscapina, oms, oms argentina, oms mexico, oseltamivir, oxolamina, pandemia, pandemia de influenza, pandemia definicion, pandemia significado, pandemia y epidemia, pandemias, pandemic, pandemica, para calmar la tos, para gripe, para la gripe, para la tos, para la tos seca, para tos seca, porcina, porcine, prevencion de influenza, prevencion de la gripe, propiedades del alcanfor, que es la gripe, que es la gripe ah1n1, que es la influenza, que es la pandemia, que es pandemia, que es una pandemia, que significa pandemia, que tomar para la tos, qura, qura plus, relenza, remedio casero para la tos, remedio para la gripe, remedio para la tos, remedios caseros para la tos, resfriado, rimivat, rino b, rino b nebu, rinofaringitis, salbutamol, sanitaria, significado de pandemia, sincicial, sintomas de fiebre, sintomas de gripe, sintomas de gripe a, sintomas de influenza, sintomas de influenza h1n1, 3m 8210, sintomas de la fiebre, sintomas de la gripe, sintomas de la gripe a, sintomas de la influenza, sintomas fiebre, sintomas gripe, sintomas gripe a, sintomas h1n1, sintomas influenza, sinusitis, swine, swine flu, tamiflu, tapabocas, te con limon, te vick, tengo gripe, tengo mucha tos, tengo tos, tos, tos con, tos con flema, tos con flemas, tos de perro, tos en bebes, tos en ninos, tos seca, tos y mocos, tratamiento de influenza, tratamiento de la gripe, tratamiento influenza, tratamiento para la gripe, trietanolamina, uso de cubrebocas, vacuna contra la gripe, vacuna influenza, vacuna para la influenza, vaporub, vick vaporub, virus ah1n1, virus de gripe, virus de influenza, virus de la gripe, virus de la influenza, virus gripe, virus h1n1, virus influenza, virus sincicial, world health organization, zanamivir

Table S5: Brazil search term list.

a gripe, a gripe h1n1, a h1n1, a1n1, ah1, ah1n1, atrovent inalação, campanha da gripe, campanha gripe, combater a gripe, contra gripe, estou com gripe, flu vaccine, gripa, gripe, gripe a, gripe comum, gripe curitiba, gripe em crianas, gripe h1, gripe h1n1, gripe hn1, gripe influenza, gripe mata, gripe sintomas, gripe suina h1n1, gripe suina no brasil, gripe vacina, gripe virus, grupo de risco, h1h1, h1m1, h1n, h1n1, h1n1 gripe, h1n1 gripe suina, h1n1 influenza, h1n1 no brasil, h1n1 prevenção, h1n1 virus, h1n1 wikipedia, h1n2, h1ni, hin1, hni, imunoclin, imunoclin curitiba, influenza, influenza h1n1, influenza vacina, n1h1, os sintomas da gripe, oseltamivir, oseltamivir bula, pneumo 23, quais os sintomas da gripe, quais sintomas da gripe, quais sção os sintomas da gripe, reação da vacina da gripe, reação vacina gripe, reações da vacina da gripe, resfriado e gripe, sintoma da gripe, sintomas da gripe, sintomas de gripe, sintomas gripe h1n1, sobre h1n1, tamiflu, tamiflu bula, tipos de gripe, tratamento para gripe, vacina contra a gripe, vacina contra gripe, vacina contra gripe h1n1, vacina contra influenza, vacina contra pneumonia, vacina da gripe, vacina da gripe h1n1, vacina de gripe, vacina de pneumonia, vacina gripe, vacina influenza, vacina para gripe, vacina pneumonia, vacinas contra gripe, vacinas da gripe, vacinas gripe, vacinas para gripe, vacinas particulares, vacinaao contra a gripe, vacinação contra gripe, vacinação da gripe, vacinação gripe, vacinação gripe.1, virus h1n1, virus h1n1

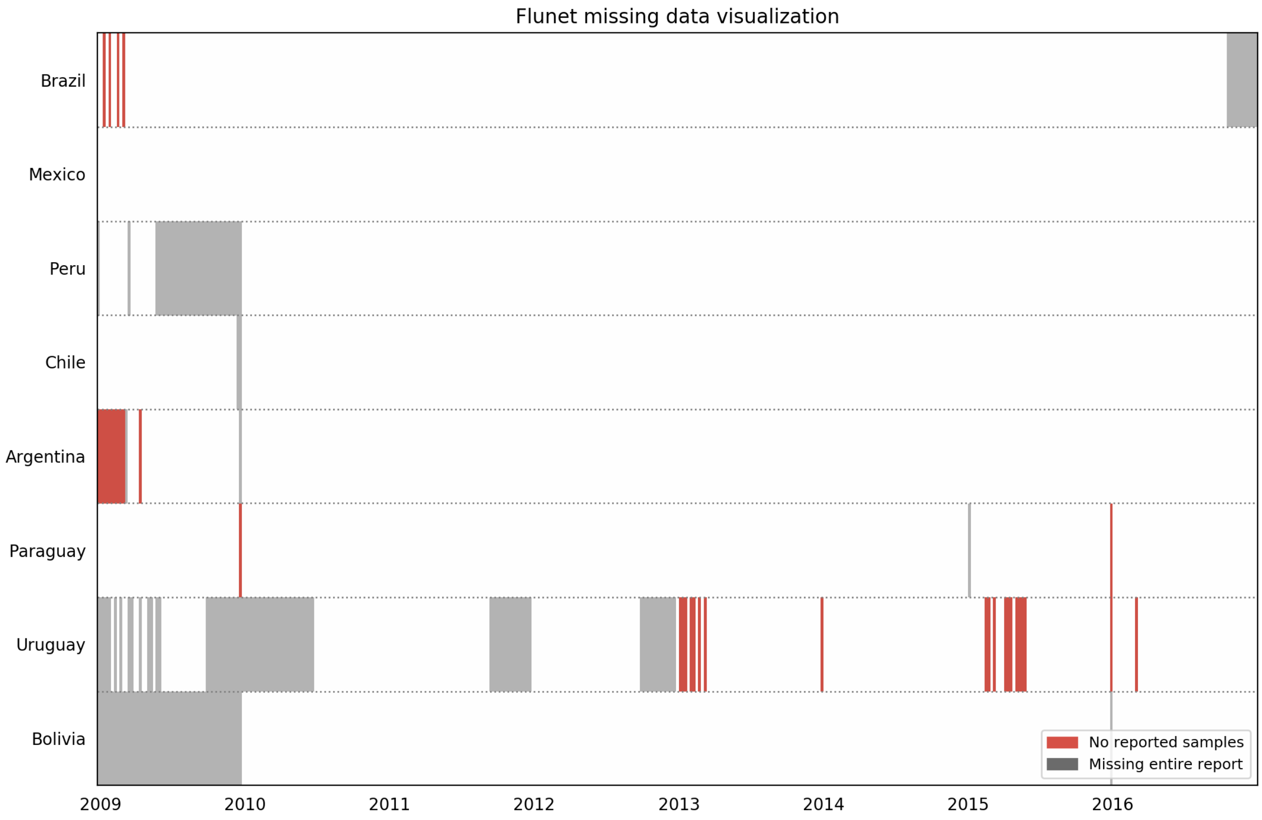

Figure S1: Heatmap displaying the report availability per country. For each row, red and gray vertical lines represent weeks where FluNet's NPS report was either missing or had no activity.

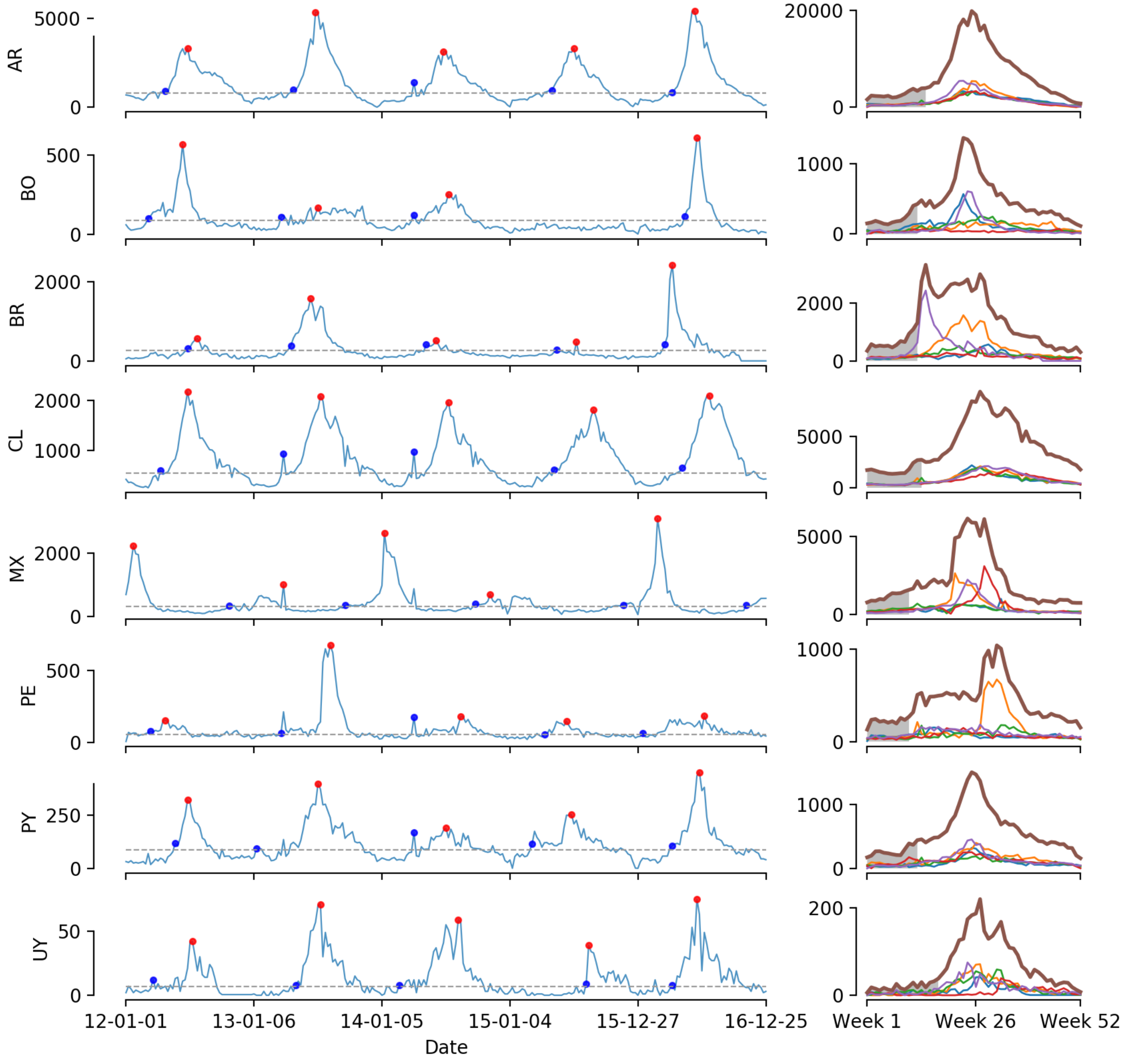

Figure S2: Left: NPS case counts containing the outbreak peaks (red) and onsets (blue) for each country. The gray line represents the threshold that divides low activity from outbreak activity. Right: Stacked timeseries for each country. The sum of the NPS curve over the gray filled area length represents 10% of the total sum of the stacked timeseries.

Table S6: Onset timing ($\Delta O = O_{model} - O_{observed}$) and Peak timing ($\Delta P = P_{model} - P_{observed}$) for each country. Positive integers indicate week delays while negative integers indicate weeks ahead of time. A "–" mark in the left table corresponds to a date where a model was not able to predict the corresponding onset or that it predicted the onset more than 4 weeks away.

Country	Onset date	ΔO_{ARGO}	ΔO_{AR}	Peak date	ΔP_{ARGO}	ΔP_{AR}
Argentina	4/22/12	-1	-1	6/24/12	1	1
	4/21/13	0	0	6/23/13	2	2
	3/30/14	-1	1	6/22/14	1	1
	4/26/15	-2	-2	6/28/15	1	-1
	4/10/16	0	0	6/5/16	0	0
Bolivia	3/4/12	–	–	6/10/12	1	1
	3/17/13	0	0	6/30/13	0	0
	3/30/14	1	1	7/6/14	4	4
	5/8/16	1	1	6/12/16	0	1
Brazil	6/24/12	-1	2	7/22/12	1	1
	4/14/13	0	0	6/9/13	1	1
	5/4/14	-3	-3	6/1/14	1	1
	5/10/15	0	1	7/5/15	1	1
	3/13/16	0	2	4/3/16	0	1
Chile	4/8/12	-1	1	6/24/12	1	1
	3/24/13	0	0	7/7/13	-1	1
	3/30/14	-1	-1	7/6/14	1	0
	5/3/15	0	0	8/23/15	-3	1
	5/1/16	-1	-1	7/17/16	-1	1
Mexico	10/21/12	1	1	1/22/12	1	1
	9/15/13	1	1	3/24/13	1	1
	9/21/14	-2	-2	1/5/14	2	1
	11/15/15	1	2	11/2/14	0	0
	10/30/16	1	1	2/21/16	1	1
Peru	3/11/12	–	–	4/22/12	0	0
	3/17/13	-1	1	8/4/13	1	1
	3/30/14	0	0	8/10/14	1	1
	4/19/15	0	-2	6/7/15	1	1
	1/10/16	-1	1	7/3/16	0	0
Paraguay	5/20/12	1	1	6/24/12	1	1
	1/6/13	1	1	6/30/13	1	1
	3/30/14	-1	-1	6/29/14	0	0
	3/1/15	1	1	6/21/15	0	0
	4/3/16	0	0	6/19/16	0	1
Uruguay	3/18/12	–	–	6/24/12	1	-3
	4/28/13	2	1	7/7/13	-4	1
	2/16/14	2	1	8/3/14	1	1
	8/2/15	1	1	8/9/15	1	1
	4/3/16	3	3	6/12/16	-3	1

Time series and coefficient heatmaps

The following set of figures displays the NPS curve as reported by the World Health Organization (black), along with the NPS estimates generated by ARGO (red), AR52 (dotted gray), and GFT (blue). Below the NPS curve are 2 error curves, which display the prediction error ($\text{Error}_t = \hat{y}_t - y_t$) and the percent error relative to the NPS value ($\%\text{Error}_t = \frac{\text{Error}_\text{t}}{y_t} = \frac{\hat{y}_t - y_t}{y_t}$). Finally, we show heatmaps representing the values of the ARGO coefficients involved in each weekly prediction.

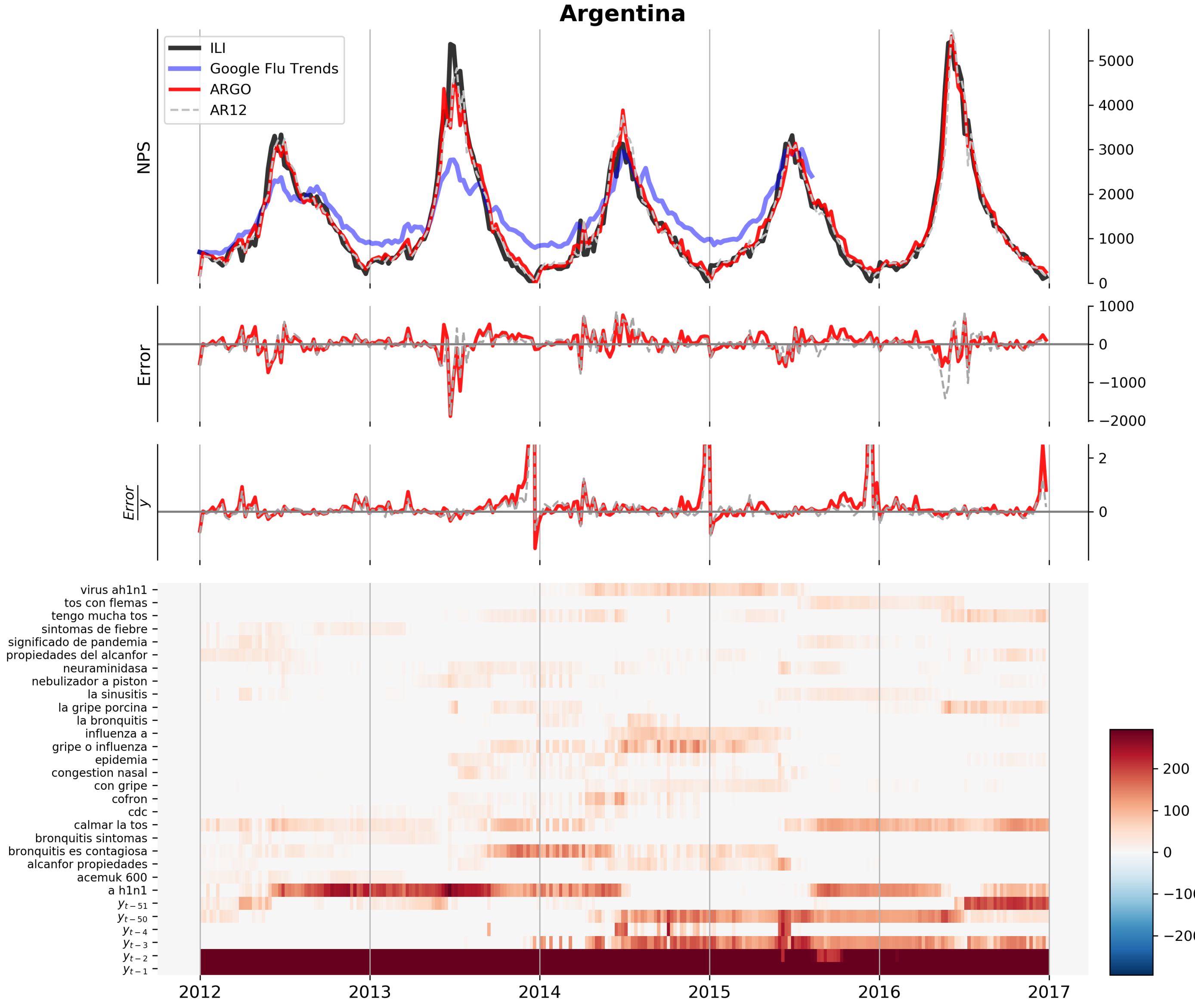

Argentina
ILI
Google Flu Trends
ARGO
AR12
NPS
5000
4000
3000
2000
1000
0
Error
1000
0
−1000
−2000
Error/y
2
0
virus ah1n1
tos con flemas
tengo mucha tos
sintomas de fiebre
significado de pandemia
propiedades del alcanfor
neuraminidasa
nebulizador a piston
la sinusitis
la gripe porcina
la bronquitis
influenza a
gripe o influenza
epidemia
congestion nasal
con gripe
cofron
cdc
calmar la tos
bronquitis sintomas
bronquitis es contagiosa
alcanfor propiedades
acemuk 600
a h1n1
y_{t-51}
y_{t-50}
y_{t-4}
y_{t-3}
y_{t-2}
y_{t-1}
200
100
0
−100
−200
2012
2013
2014
2015
2016
2017

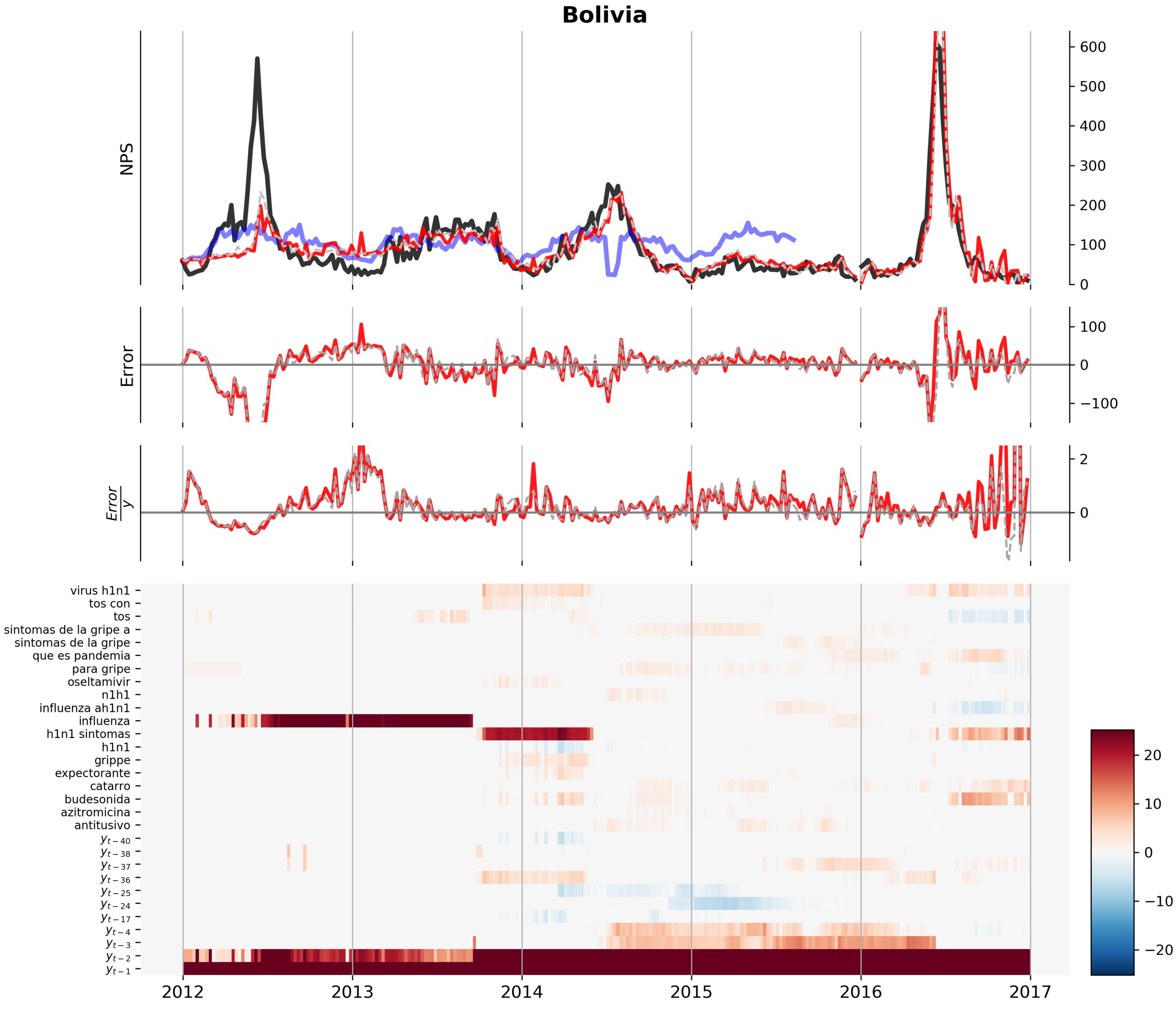
Bolivia
NPS
Error
$\frac{Error}{y}$
virus h1n1
tos con
tos
sintomas de la gripe a
sintomas de la gripe
que es pandemia
para gripe
oseltamivir
n1h1
influenza ah1n1
influenza
h1n1 sintomas
h1n1
grippe
expectorante
catarro
budesonida
azitromicina
antitusivo
y_{t-40}
y_{t-38}
y_{t-37}
y_{t-36}
y_{t-25}
y_{t-24}
y_{t-17}
y_{t-4}
y_{t-3}
y_{t-2}
y_{t-1}
2012
2013
2014
2015
2016
2017
0
100
200
300
400
500
600
−100
2
−20
−10
10
20

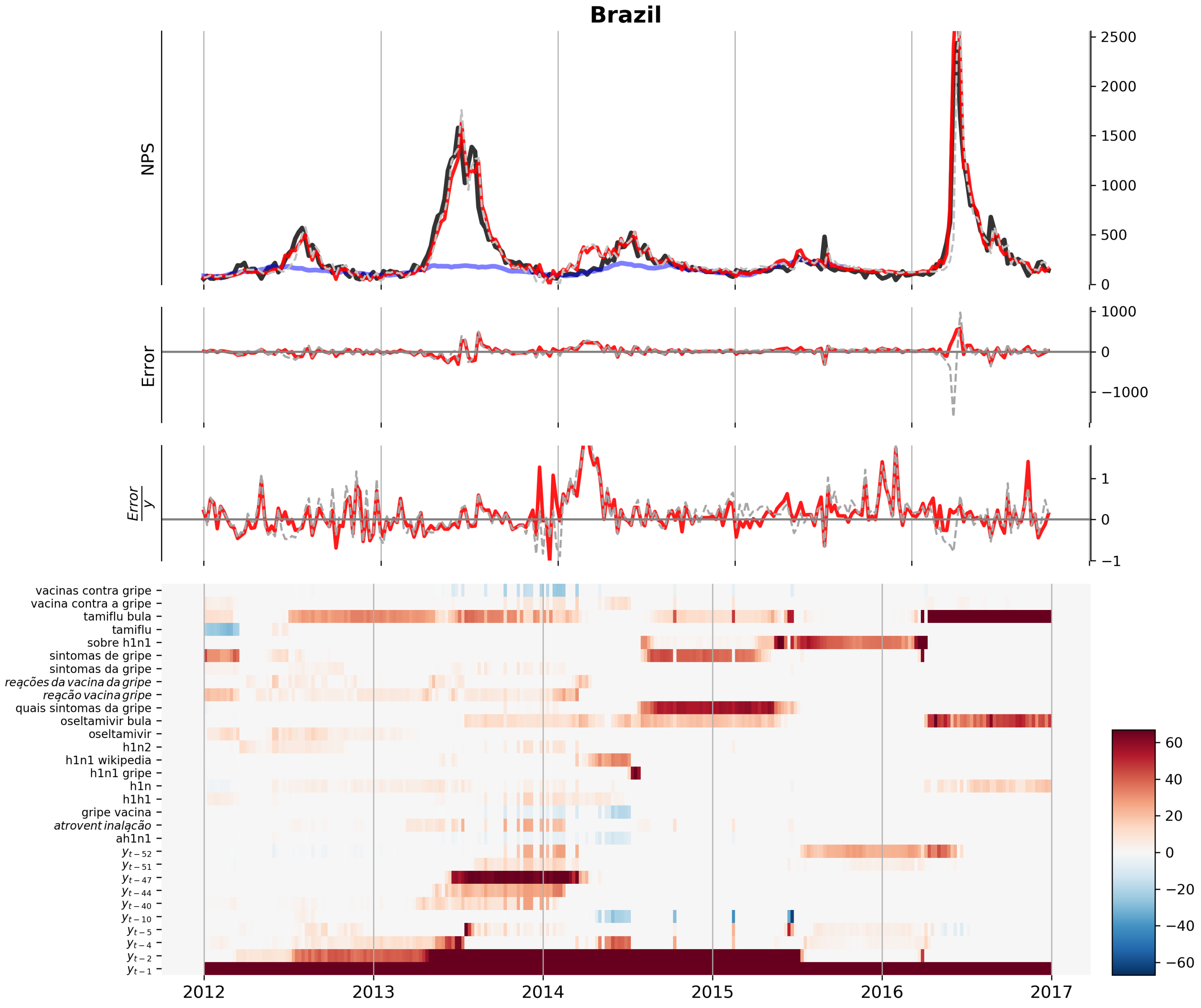
Brazil
NPS
Error
$\frac{Error}{y}$
vacinas contra gripe
vacina contra a gripe
tamiflu bula
tamiflu
sobre h1n1
sintomas de gripe
sintomas da gripe
reações da vacina da gripe
reação vacina gripe
quais sintomas da gripe
oseltamivir bula
oseltamivir
h1n2
h1n1 wikipedia
h1n1 gripe
h1n
h1h1
gripe vacina
atrovent inalação
ah1n1
y_{t-52}
y_{t-51}
y_{t-47}
y_{t-44}
y_{t-40}
y_{t-10}
y_{t-5}
y_{t-4}
y_{t-2}
y_{t-1}
2012
2013
2014
2015
2016
2017

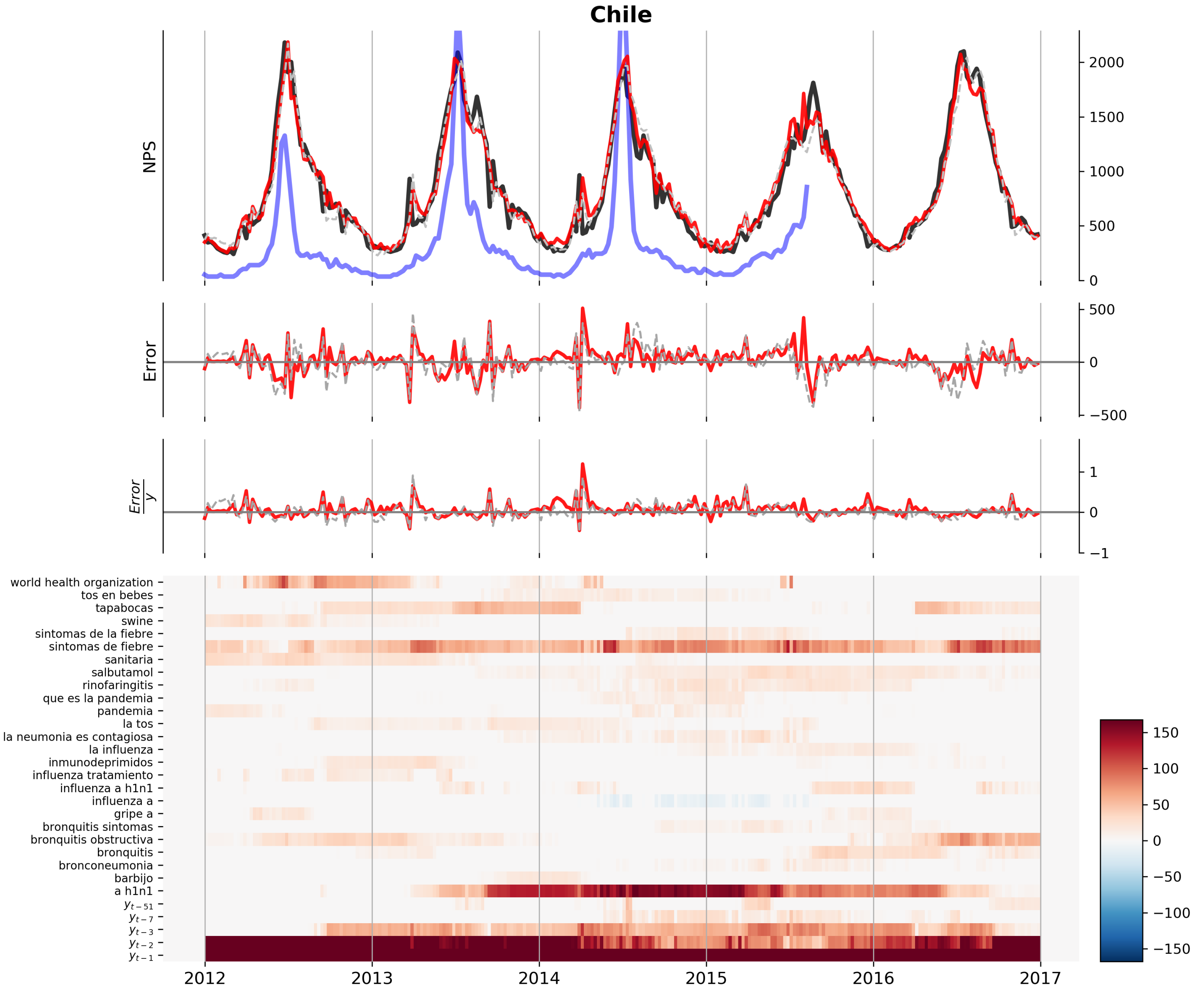

Chile
NPS
2000
1500
1000
500
0
Error
500
0
−500
$\frac{Error}{y}$
1
0
−1
world health organization
tos en bebes
tapabocas
swine
sintomas de la fiebre
sintomas de fiebre
sanitaria
salbutamol
rinofaringitis
que es la pandemia
pandemia
la tos
la neumonia es contagiosa
la influenza
inmunodeprimidos
influenza tratamiento
influenza a h1n1
influenza a
gripe a
bronquitis sintomas
bronquitis obstructiva
bronquitis
bronconeumonia
barbijo
a h1n1
y_{t-51}
y_{t-7}
y_{t-3}
y_{t-2}
y_{t-1}
150
100
50
0
−50
−100
−150
2012
2013
2014
2015
2016
2017

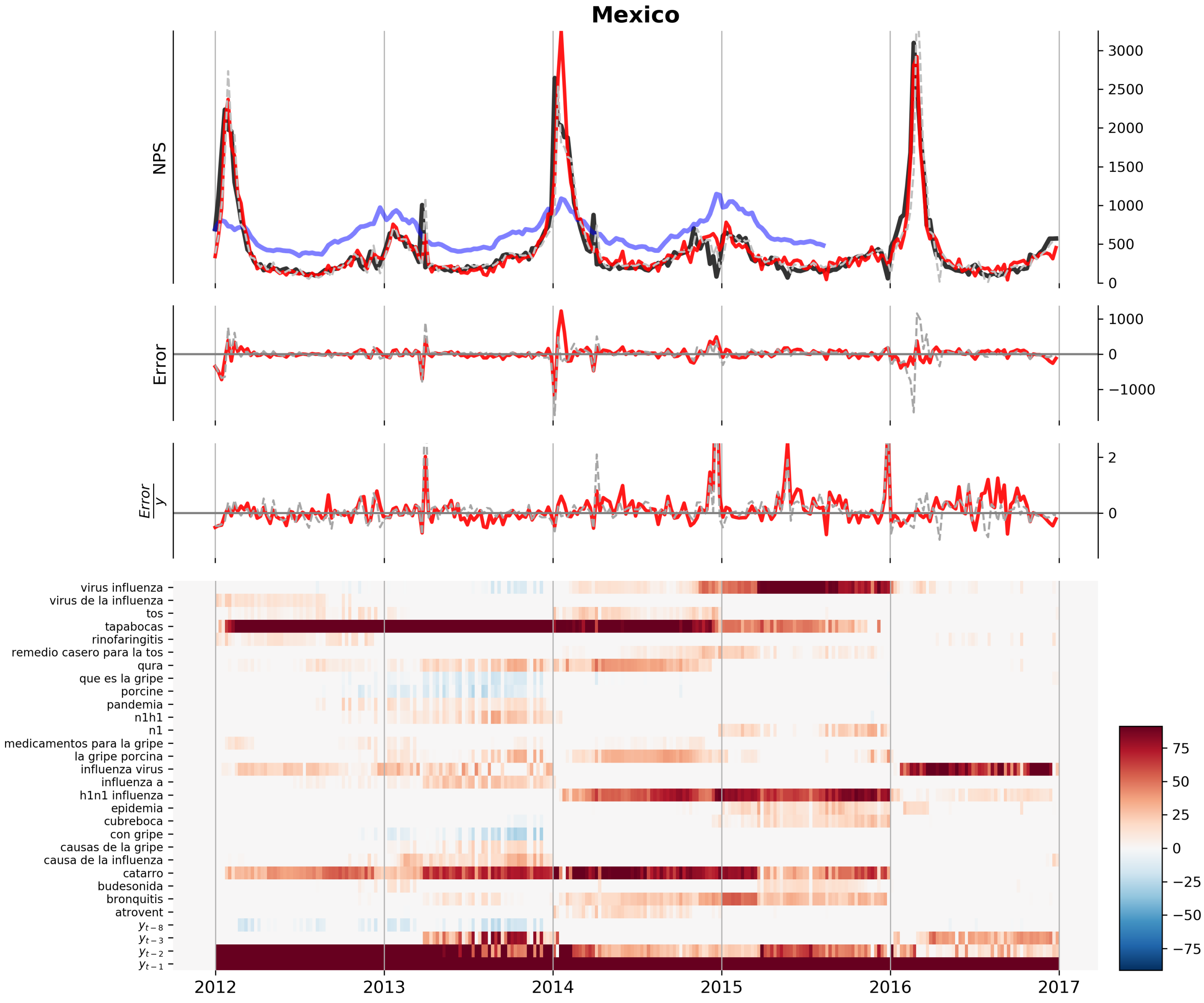
Mexico
NPS
Error
$\frac{Error}{y}$
3000
2500
2000
1500
1000
500
0
1000
0
−1000
2
0
virus influenza
virus de la influenza
tos
tapabocas
rinofaringitis
remedio casero para la tos
qura
que es la gripe
porcine
pandemia
n1h1
n1
medicamentos para la gripe
la gripe porcina
influenza virus
influenza a
h1n1 influenza
epidemia
cubreboca
con gripe
causas de la gripe
causa de la influenza
catarro
budesonida
bronquitis
atrovent
y_{t-8}
y_{t-3}
y_{t-2}
y_{t-1}
2012
2013
2014
2015
2016
2017
75
50
25
0
−25
−50
−75

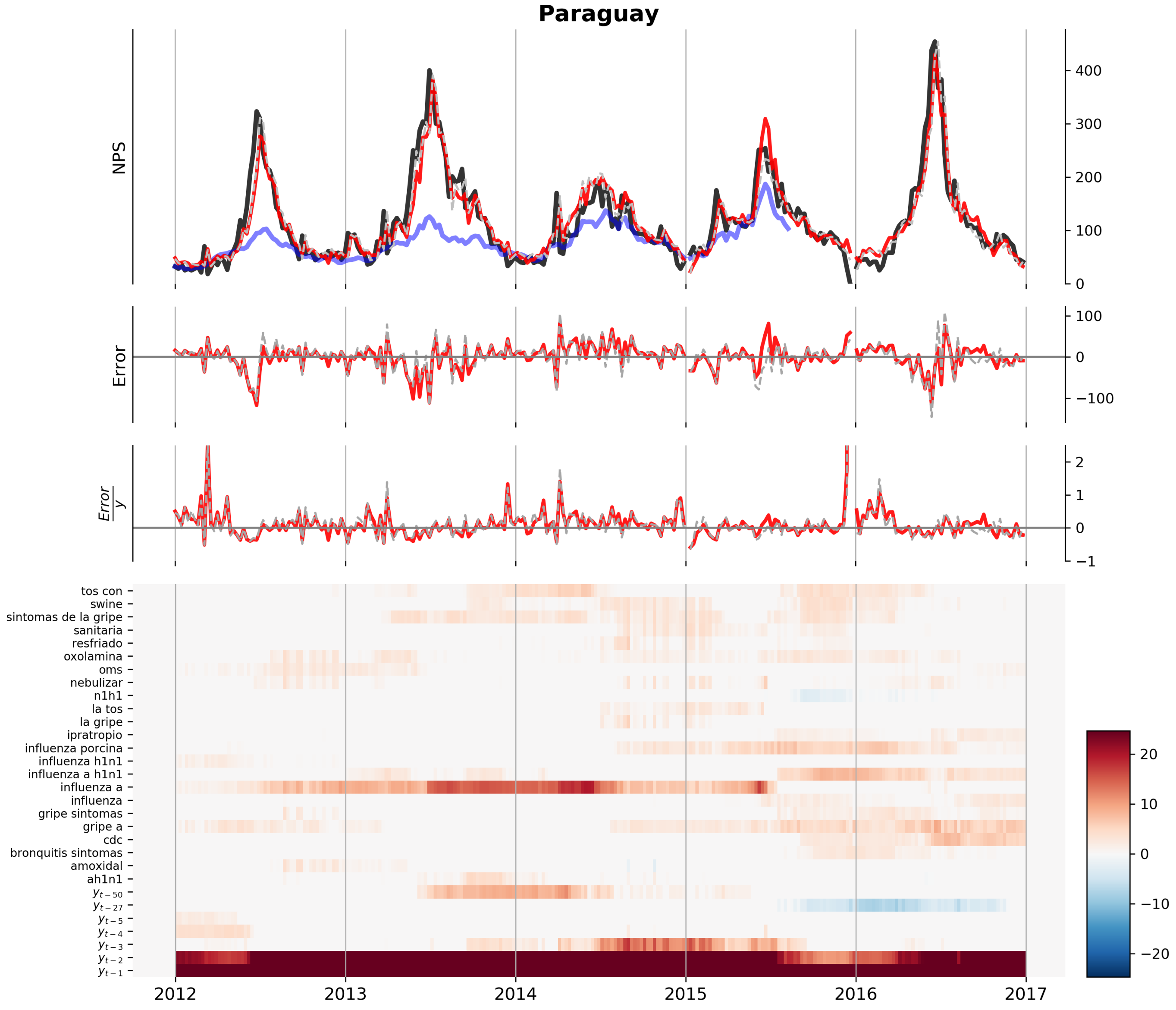

Paraguay
NPS
400
300
200
100
0
Error
100
0
−100
$\frac{Error}{y}$
2
1
0
−1
tos con
swine
sintomas de la gripe
sanitaria
resfriado
oxolamina
oms
nebulizar
n1h1
la tos
la gripe
ipratropio
influenza porcina
influenza h1n1
influenza a h1n1
influenza a
influenza
gripe sintomas
gripe a
cdc
bronquitis sintomas
amoxidal
ah1n1
y_{t-50}
y_{t-27}
y_{t-5}
y_{t-4}
y_{t-3}
y_{t-2}
y_{t-1}
2012
2013
2014
2015
2016
2017
20
10
0
−10
−20

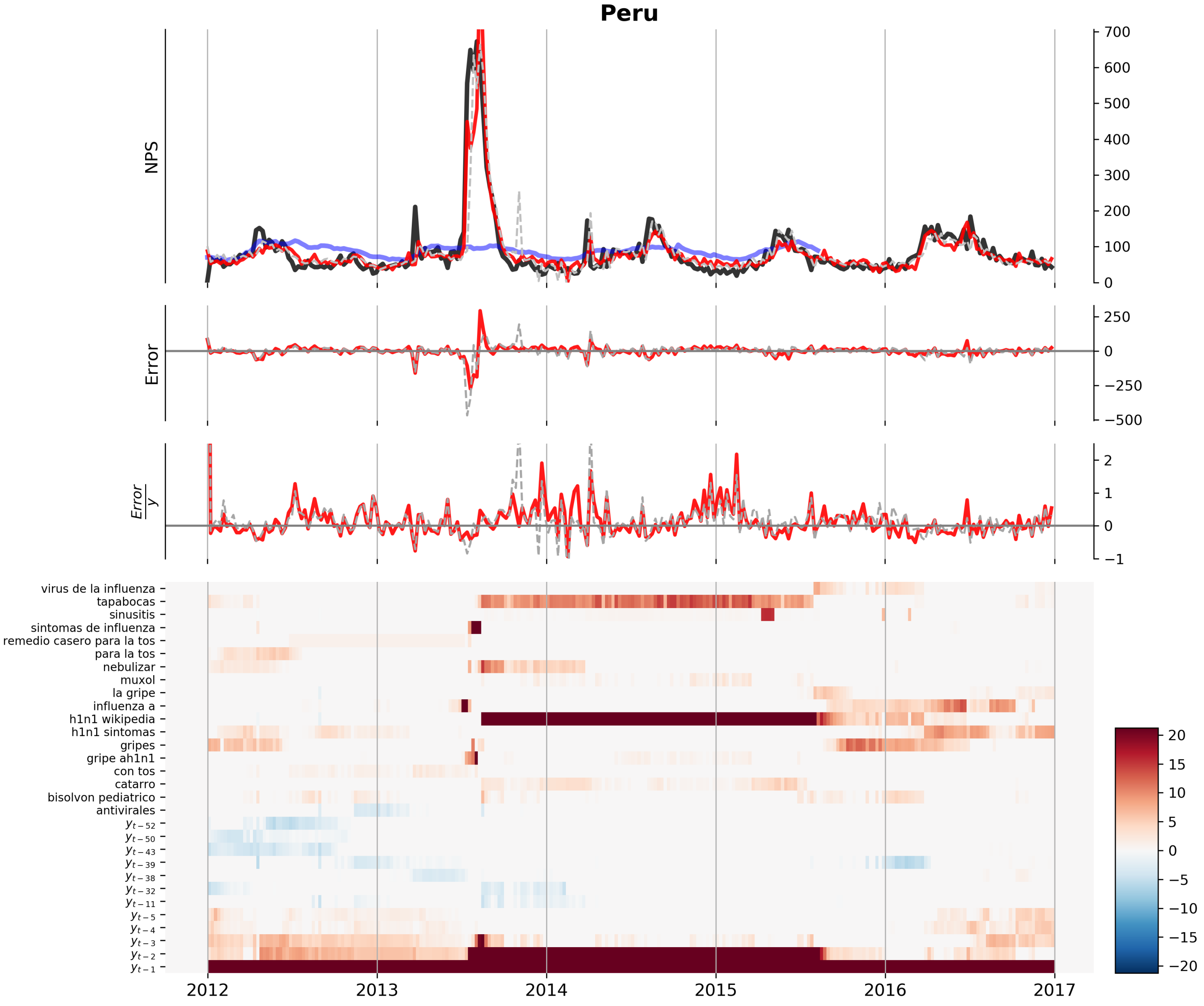

Peru
NPS
Error
$\frac{Error}{y}$
virus de la influenza
tapabocas
sinusitis
sintomas de influenza
remedio casero para la tos
para la tos
nebulizar
muxol
la gripe
influenza a
h1n1 wikipedia
h1n1 sintomas
gripes
gripe ah1n1
con tos
catarro
bisolvon pediatrico
antivirales
y_{t-52}
y_{t-50}
y_{t-43}
y_{t-39}
y_{t-38}
y_{t-32}
y_{t-11}
y_{t-5}
y_{t-4}
y_{t-3}
y_{t-2}
y_{t-1}
2012
2013
2014
2015
2016
2017

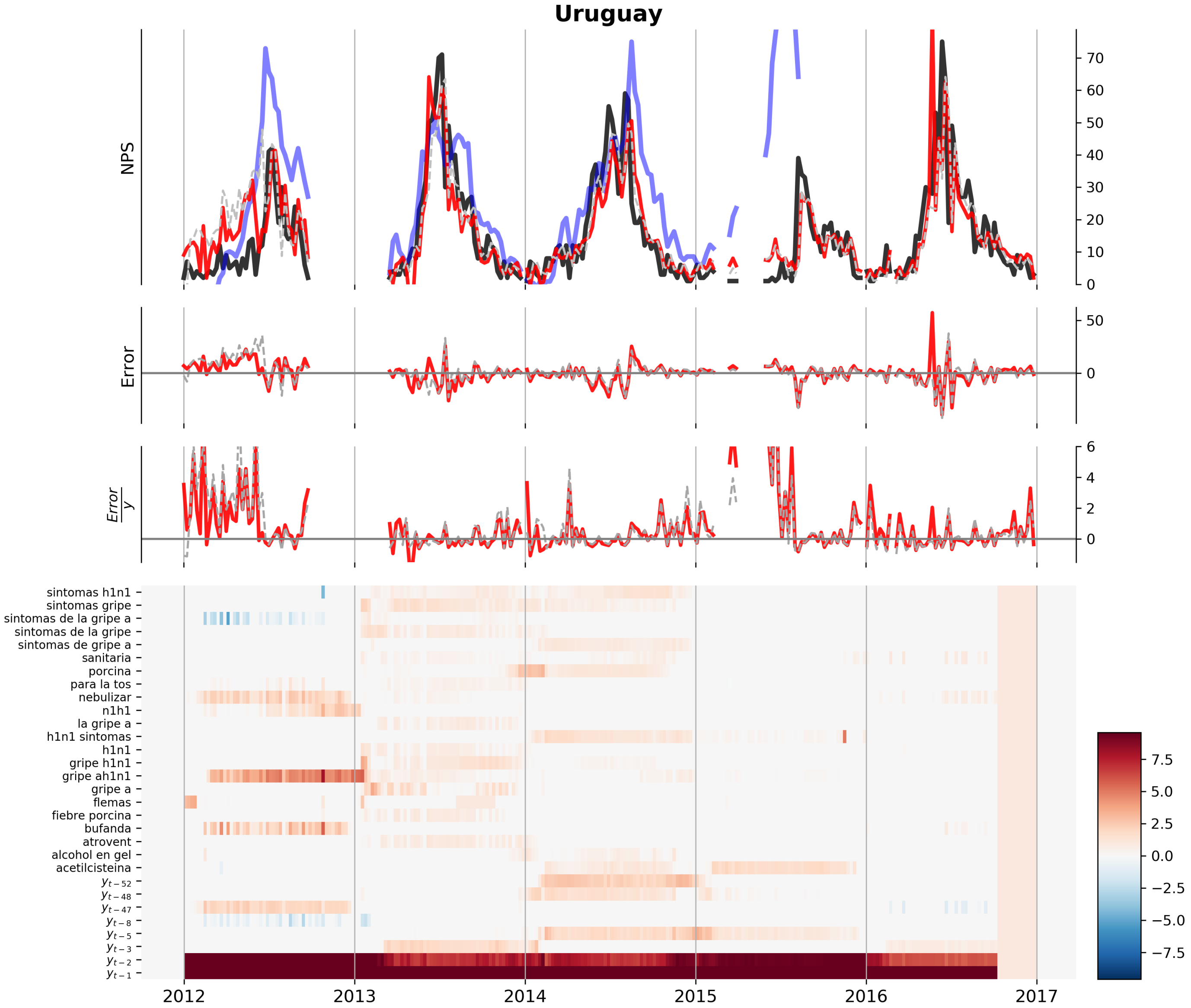

Uruguay
NPS
Error
$\frac{Error}{y}$
sintomas h1n1
sintomas gripe
sintomas de la gripe a
sintomas de la gripe
sintomas de gripe a
sanitaria
porcina
para la tos
nebulizar
n1h1
la gripe a
h1n1 sintomas
h1n1
gripe h1n1
gripe ah1n1
gripe a
flemas
fiebre porcina
bufanda
atrovent
alcohol en gel
acetilcisteina
y_{t-52}
y_{t-48}
y_{t-47}
y_{t-8}
y_{t-5}
y_{t-3}
y_{t-2}
y_{t-1}
2012
2013
2014
2015
2016
2017
